# Klotho and Aminopeptidases as Early Biomarkers of Renal Injury in Zucker Obese Rats

**DOI:** 10.3389/fphys.2018.01599

**Published:** 2018-11-13

**Authors:** Sebastián Montoro-Molina, Antonio López-Carmona, Andrés Quesada, Francisco O’Valle, Natividad Martín-Morales, Antonio Osuna, Félix Vargas, Rosemary Wangensteen

**Affiliations:** ^1^Departamento de Fisiología, Facultad de Medicina, Universidad de Granada, Granada, Spain; ^2^Departamento de Ciencias de la Salud, Universidad de Jaén, Jaén, Spain; ^3^Servicio de Nefrología, Unidad Experimental, Hospital Universitario Virgen de las Nieves, Granada, Spain; ^4^Departamento de Anatomía Patológica, Instituto de Biopatología y Medicina Regenerativa (IBIMER), Facultad de Medicina, Universidad de Granada, Granada, Spain; ^5^Instituto de Investigación Biosanitaria de Granada (ibs.GRANADA), Granada, Spain

**Keywords:** Zucker rats, aminopeptidases, Klotho, hydroxyproline, renal injury

## Abstract

The aim of this study was to investigate if urinary glutamyl aminopeptidase (GluAp), alanyl aminopeptidase (AlaAp), Klotho and hydroxyproline can be considered as potential biomarkers of renal injury and fibrosis in an experimental model of obesity. Male Zucker lean (ZL) and obese (ZO) rats were studied from 2 to 8 months old. Kidneys from ZO rats at the end of the study (8 months old) developed mild focal and segmental glomerulosclerosis as well as moderate tubulointerstitial injury. Urinary excretion of Klotho was higher in ZO rats at 2, 5, and 8 months of study, plasma Klotho levels were reduced and protein abundance of Klotho in renal tissue was similar in ZL and ZO rats. GluAp and AlaAp urinary activities were also increased in ZO rats throughout the time-course study. ZO rats showed an augmentation of hydroxyproline content in renal tissue and a significant increase of tubulointerstitial fibrosis. Correlation studies demonstrated that GluAp, AlaAp, and Klotho are early diagnostic markers of renal lesions in Zucker obese rats. Proteinuria and hydroxyproline can be considered delayed diagnostic markers because their contribution to diagnosis starts later. Another relevant result is that GluAp, AlaAp, and Klotho are related not only with diagnosis but also with prognosis of renal lesions in Zucker obese rats. Moreover, strong predictive correlations of aminopeptidasic activities with the percentage of renal fibrosis or with renal hydroxyproline content at the end of the experiment were observed, indicating that an early increased excretion of these markers is related with a higher later extent of fibrosis in Zucker obese rats. In conclusion, GluAp, AlaAp, and Klotho are early diagnostic markers that are also related with the extent of renal fibrosis in Zucker obese rats. Therefore, they have a potential use not only in diagnosis, but also in prognosis of obesity-associated renal lesions.

## Introduction

The Zucker obese rat is an experimental model that mimicked diabetes type II in human (de Artinano and Castro, 2009). This model results from an autosomal recessive mutation of the *fa*-gene, en coding the leptin receptor; and courses with obesity, insulin resistance, dyslipidemia, mild glucose intolerance and renal injury, whereas the Zucker lean rat is lacking of leptin receptor mutation and of metabolic and renal abnormalities.

Renal injury is characterized by an increased time related proteinuria, focal segmental glomerulosclerosis and tubulointerstitial damage that culminates ultimately in renal failure and death (Kasiske et al., 1985, 1992; Magil, 1995; Coimbra et al., 2000; Gassler et al., 2001; D’Agati et al., 2016).

Renal clinical practice requires highly sensitive and specific diagnostic and prognostic biomarkers for acute and chronic kidney disease (CKD). The early identification of renal disease onset and risk stratification in CKD progression are essential for early treatment of patients to ameliorate their comorbidity burden, particularly cardiovascular disease (CVD), and prevent the development of end stage renal disease (ESRD).

Enzymes released from damaged tubular cells and excreted into urine are the most promising biomarkers for an early detection of kidney injury. They have an obvious diagnostic benefit because their measurements may provide detailed information about the nature, size and site of the damage to tubular cells and their possible necrosis or dysfunction (Lisowska-Myjak, 2010). Alanyl aminopeptidase (AlaAp, EC3.4.11.2), and glutamyl aminopeptidase (GluAp, EC 3.4.11.7) are present in the renal tubular cells (Kenny and Maroux, 1982; Song et al., 1994; Albiston et al., 2011) and exert its aminopeptidasic activity in the renal angiotensin II metabolism, factor that is activated in renal injury of diabetic rodents and humans (Gagliardini et al., 2013).

In previous works, we determined the activity of these aminopeptidases as an index of renal damage in hypertensive hyperthyroid rats under normal, high and low saline intake (Pérez-Abud et al., 2011) and in cisplatin treated rats (Quesada et al., 2012). Urinary aminopeptidases, mainly AlaAp and GluAp, were revealed as early and predictive biomarkers of renal injury severity (Quesada et al., 2012; Montoro-Molina et al., 2015). More recently, we have reported that GluAp in microvesicular and exosomal fractions of urine is also related with renal dysfunction in cisplatin-induced nephrotoxicity and that serve to detect proximal tubular damage independently of glomerular filtration status (Quesada et al., 2017).

Schnackenberg et al. (2012) observed that in a model of melamine and cyanuric acid nephrotoxicity, urinary hydroxyproline was increased in male and female Fischer F344 rats; and that rats with the highest levels of urinary hydroxyproline exhibited renal fibrosis. These authors concluded that hydroxyproline may be a non-invasive urinary biomarker for detection of acute kidney injury associated with renal fibrosis.

Klotho is a transmembrane protein that is expressed mainly in the kidney. Parathyroid gland and choroid plexus are also sites of abundant Klotho expression (Kuro-o et al., 1997; Erben, 2016). The soluble extracellular domain is cleaved, presumably from renal cells, and secreted into the blood or other biological fluids (Ito et al., 2000). Thus, Klotho can be found in two forms tisular and secreted or soluble αKlotho. The kidney is probably the major source of the soluble form, since serum soluble Klotho was reduced by about 80% in kidney-specific Klotho knockout mice (Lindberg et al., 2014).

In the kidney, Klotho is expressed in the distal convoluted tubules, in the proximal convoluted tubule (Hu et al., 2010a; Ide et al., 2016) and the inner medullary collecting duct (Mitobe et al., 2005). Klotho mRNA expression in rat kidney is down regulated in various animal models of vascular and metabolic diseases such as hypertension, hyperlipidemia, renal failure, and inflammatory stress (Nagai et al., 2000).

Under physiological conditions, the kidney has dual effects over soluble αKlotho homeostasis, producing and cleaving αKlotho from the membrane of the renal tubular epithelial cells into the circulation and removing αKlotho from the blood into the tubular urinary lumen through transcytosis to cross renal tubules (Hu et al., 2016).

Soluble αKlotho is the main functional form in the circulation (Hu et al., 2016), cerebrospinal fluid (Semba et al., 2014; Chen et al., 2015), and urine (Hu et al., 2010a, 2011) acting as an endocrine factor on distant organs (Hu et al., 2013, 2015). Soluble Klotho is markedly decreased in CKD and ESRD (Lu and Hu, 2017). It has been proposed as a novel, early and sensitive biomarker for renal and CVDs and that can also serve as a predictor for risk of CVD and mortality in both CKD patients and the general population (Shimamura et al., 2012; Scholze et al., 2014). In CKD patients, the magnitude of decrease in urinary Klotho was correlated with the severity of decline in GFR (Hu et al., 2011).

In this work we characterized the histopathological manifestations of obesity –induced renal injury in obese Zucker (ZO) rats; and investigated the urinary enzymatic activities of AlaAp and GluAp, plasma, urinary and renal Klotho levels and urinary and renal hydroxyproline as potential biomarkers of the renal injury observed in ZO rats.

## Materials and Methods

### Ethics Statement

All experimental procedures were carried out in accordance with the principles of the Basel Declaration and recommendations of European Union Guidelines to the Care and Use of Laboratory Animals. The protocol was approved by the Ethical Committee of the University of Jaén and authorized by Junta de Andalucía with the approval identification number 450-5297 (02/21/2014).

### Animals

Male Zucker lean and obese rats (ZL and ZO, *n* = 10 each group) at 2 months old were purchased from Charles River Laboratories (Wilmington, MA, United States) and studied up to 8 months old. These rats were kept in a room maintained at 24 ± 1°C and humidity of 55 ± 10%, with a 12 h light/dark cycle and had free access to rat chow and tap water.

### Experimental Protocol

All rats were weighed and housed in metabolic cages (Panlab, Barcelona, Spain) once a month during 24 h with free access to food and drinking. Food and fluid intakes were measured and 24 h urine samples were collected. Urine samples were centrifuged for 15 min at 1000 *g* and frozen at -80°C until assay. We measured monthly urine volume, proteinuria, glucosuria, creatinine, hydroxyproline, GluAp, and AlaAp activities. Urinary Klotho was measured at 2, 5, and 8 months old.

After completion of the experimental period (at 8 months old), rats were anesthetized with pentobarbital, 50 mg/kg, i.p. Blood samples were then drawn by abdominal aortic puncture to determine plasma variables. Blood samples were centrifuged for 15 min at 1000 *g* and stored at -80°C. Finally, rats were killed by pentobarbital overdose (200 mg/kg, i.p.), and kidneys and heart were quickly removed and weighed.

Plasma variables were: glucose, urea, creatinine and Klotho. Tibial length was measured to normalize heart and kidney weight, since body weight cannot be used for this purpose in the present experimental setting. One kidney was fixed in 10% neutral-buffered formaldehyde solution for 48 h and subsequently placed in 70% ethanol for histological studies.

### Analytical Procedures

Proteinuria and urine creatinine were determined in urine samples by an autoanalyzer Spin120. Plasma creatinine, urea and glucose were also measured in this instrument. Reagents for proteinuria, urea, and creatinine-Jaffé method were provided by Spinreact (Barcelona, Spain). Plasma and urinary Klotho levels were determined with an enzyme immunoassay kit, Bioassay Technology Laboratory (Shanghai, China). Renal and urinary hydroxyproline was measured with a Hydroxyproline Assay Kit (Sigma-Aldrich, Madrid, Spain). Protein content in renal tissue was analyzed with DC Bio-Rad protein assay (Madrid, Spain).

### Measurement of Hydroxyproline in Urine and Renal Tissue

Hundred microliter of urine sample or 100 μl of renal tissue (100 mg/ml in water) were hydrolyzed with the same volume of 37% HCl at 120°C overnight. 5 mg of activated charcoal were added to samples, mixed and centrifuged at 13,000 *g* for 2 min. 50 μl of each supernatant were transferred to a 69-well plate and dried in a 60°C oven until dryness. 100 μl of chloramine T/oxidation buffer mixture, 50 μl of 4-(dimethylamino) benzaldehyde concentrate and 50 μl of perchloric acid/isopropanol solution were added to each well and plates were heated for 90 min at 60°C. Absorbance was measured at 560 nm. The amount of hydroxyproline in each well was calculated from a standard curve of hydroxyproline containing 0–1 μg/well.

### Western Blotting of Klotho in Renal Tissue

Kidneys were homogenized using RIPA lysis cell buffer containing protease inhibitors cocktail (05892791001, Roche, Germany). 30 μg of protein were subjected to SDS-PAGE electrophoresis using 50 μL/well ready gels purchased from Bio-Rad (Madrid, Spain). Proteins were transferred to a nitrocellulose membrane which was probed with 1 μg/mL rabbit anti-Klotho-antibody (Sigma-Aldrich, Madrid, Spain), and 20 ng/mL goat anti-rabbit IgG antibody HRP-conjugated (KPL Diagnostics, Gaithersburg, MD, United States). Bands were visualized with an enhanced chemiluminescence system (ECL, Amersham, United Kingdom) in a CCD camera image system and quantified with Image J software (v 1.48) (National Institute of Health, United States).

### Measurement of Aminopeptidasic Activities

GluAp and AlaAp fluorimetric activities were determined in a kinetic fluorimetric assay using L-glutamic acid γ-2-naphthylamide or alanyl-2-naphthylamide (Sigma-Aldrich, Madrid, Spain) as substrates, respectively. 20 μl of urine, plasma or kidney homogenate were incubated during 30 min at 37°C with 80 μl of substrate solution (10 mM L-glutamic acid γ-2-naphthylamide or 10 mM alanyl-2-naphthylamide in pH 8.7 50 mM HCl-Tris). This buffer was also used to homogenate kidneys. Substrates had been previously dissolved in 1 ml of dimethyl sulfoxide and stored at -20°C. The amount of 2-naphthylamine released as a result of the aminopeptidase activities was measured fluorimetrically at an emission wavelength of 412 nm with an excitation wavelength of 345 nm, and quantified using a standard curve of 2-naphthylamine (0–200 nmol/ml). Fluorimetric data from samples and standard curve were taken each minute. Specific aminopeptidase activities were calculated from the slope of the linear portion of enzymatic assay, and expressed as nanomol of substrate hydrolyzed per minute per mg of urine creatinine (mU/mg Cr) per ml of plasma or per mg of total protein content in renal tissue.

### Histopathological Study

For conventional morphology, buffered 10% formaldehyde-fixed, paraffin-embedded longitudinal rat kidney sections in sagittal plane were deparaffinized in xylol (three passes of 5 min) and re-hydrated in ethanol of decreasing gradation (absolute, 96%, and 70%, 2 passes of 3 min, respectively). Tissue sections were stained with hematoxylin-eosin, Masson’s trichrome and periodic acid-Schiff (PAS). The presence of glomerular lesions (glomerulosclerosis, glomerular hyperplasia, mesangium increase, glomerular cyst, and capsular fibrosis) was assessed in at least 200 glomeruli. Tubulointerstitial damage (tubular vacuolation, tubular atrophy, hyaline drops, tubular casts, and chronic inflammatory infiltrate) was also studied. Injury was graded according to Shih, Hines, and Neilson on a semiquantitative scale of 0–4 (0 = normal, 0.5 = small focal areas of damage, 1 = involvement of less than 10% of the cortex, 2 = involvement of 10–25% of the cortex, 3 = involvement of 25–75% of the cortex, 4 = extensive damage involving more than 75% of the cortex) (Shih et al., 1988). The morphological study was done in blinded fashion on 4-micrometer sections with light microscopy, using the most appropriate stain for each lesion.

### Morphometrical Study

Samples were fixed in buffered 10% formalin, embedded in paraffin and serially sectioned at 5 μm thickness. Afterwards, they were stained with 1% picro Sirius red F3BA (Gurr, BDH Chemicals Ltd., Poole, United Kingdom) for image analysis quantification. To improve staining, tissue sections were kept after deparaffination for 3–5 days in 70% alcohol as mordent. Picro Sirius red stains connective fibers deep red and cell nuclei and cytoplasmatic structures light red/bright yellow (Sweat et al., 1964). To semiautomatically quantify interstitial connective tissue, 20 images of cortex per kidney were acquired using an IF 550 green optical filter with illumination intensity values slightly above those used for normal observation with a digital camera 3CCD (DP70) coupled to an Olympus BX-42 microscope (Olympus Optical Company). 20 images of corticomedullary junction per kidney were acquired using polarized light. Histologic images of kidney biopsies were convert in black and white at 8-bit intensity resolution (256 gray levels) with a global magnification of 200×, and normalized with Adobe Photoshop software (Adobe Systems Software, Ireland). To assess the fibrosis, we made a macro that included a semiautomatic thresholding of the total of the images per group of rats simultaneously with ImageJ software (v 1.48) (National Institute of Health, United States).

### Statistical Analyses

To study the time course of biological variables and urinary markers, we used a factorial ANOVA for repeated measures, taking each rat as the subject and the group (ZL or ZO) as the between-subjects factor. Interactions between factors were analyzed using Bonferroni method. We used StatGraphics Centurion XVII software.

Morphological and biological variables measured at the end of the experiment were compared using a *t*-test for the analysis of variables with normal distribution and equal variances. Welch modification of *t*-test was used for data with normal distribution and unequal variances and Mann–Whitney W (Wilcoxon) test was used to analyze the differences when data did not correspond to a normal distribution. Shapiro–Wilk test was used to analyze the normality of distributions. Differences were considered statistically significant at *p* < 0.05 level. We used StatGraphics Centurion XVII software.

Simple linear regressions were analyzed with Statgraphics Centurion XVII software. Multiple linear regressions were also analyzed with this software to establish the optimal correlation between urinary markers as independent variables and each renal lesion as dependent variable. The model of regression with best information criteria (Akaike information criteria) was selected. This model eliminates the markers that do not significantly contribute to regression, obtaining an optimal fitted model that includes all markers with a significant contribution to regression (*p* < 0.05).

For histopathological results IBM SPSS-Windows 20.0 (SPSS Inc., Chicago, IL, United States) was used for the analyses. Results are presented as mean ± standard error in the case of fibrosis, or median and interquartilic range for categorical data. *t*-test was used to compare percentage of fibrosis. Non-parametric Mann–Whitney *U*-test were used to compared morphological and histomorphometrical variables. Results were considered statistically significance when *p*-values were below 0.05.

## Results

### Time Course of Biological Variables

A comparison of the biological variables between ZO and ZL rats was performed (data not shown). The variables analyzed were: body weight, food intake, water intake, diuresis and water balance. Body weight was obviously greater in ZO with respect to ZL rats. Food intake was increased in ZO rats, whereas water intake was reduced along the study. Unexpectedly, diuresis was higher in ZO rats; and consequently, water balance was found reduced in these animals. The time course of glucosuria was not significantly different in ZO and ZL rats when expressed in concentration, but it was significantly increased from 6 to 8 months old when data were normalized by creatinine excretion.

### Morphological Variables

Final body weight was significantly increased in ZO rats by 1.35-fold when compared to ZL rats. Kidney weight and heart weight in absolute values or relative to tibial length were increased in ZO rats when compared with the ZL group (Table 1).

**Table 1:** Morphological variables in male Zucker lean (ZL) and obese (ZO) rats at 8 months old.

Groups	ZL	ZO
Body weight (g)	469.1 ± 7.04	631.6 ± 10.2^∗∗^
Tibial length (TL, cm)	5.455 ± 0.042	5.480 ± 0.025
Kidney weight (KW, g)	1.628 ± 0.074	2.027 ± 0.105^∗∗^
KW/TL (mg/cm)	298.1 ± 12.5	398.4 ± 19.3^∗∗^
Heart weight (HW, g)	1.258 ± 0.020	1.396 ± 0.032^∗^
HW/TL (mg/cm)	230.6 ± 2.88	274.7 ± 5.52^∗∗^

*Data are expressed as means ± SE final body weight; kidney weight; KW/TL, ratio kidney weight versus tibial length; HW/TL, ratio heart weight versus tibial length. ^∗^P < 0.01, ^∗∗^P < 0.001 vs. the control group (n = 10 in each group)*.

### Histopathological Results

**FIGURE 1:**
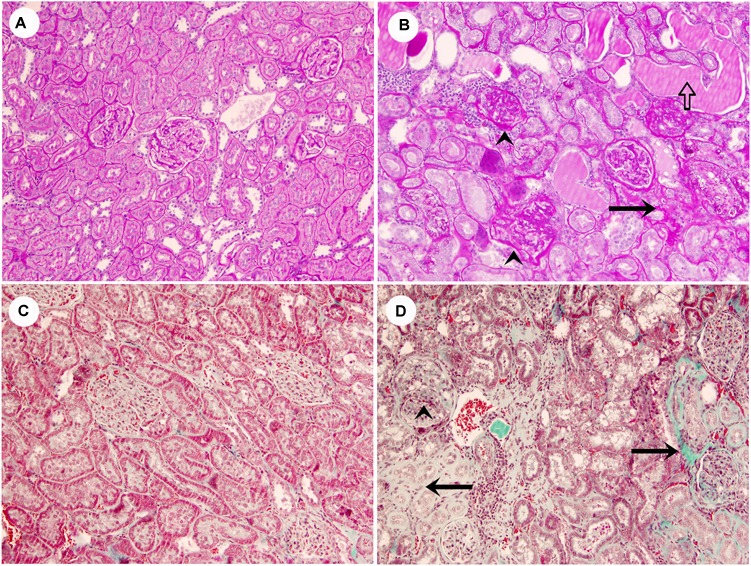
Representative images of lean rats without glomerular or tubulointerstitial injury **(A,C)**, and obese rats with segmental glomerular sclerosis (ˆ), tubular atrophy (black arrows), tubular casts (white arrow) and inflammatory infiltrate **(B,D)** (**A,B**, PAS stain; **C,D**, Masson’s trichrome stain, original magnification 10×).

Renal lesions in ZL group were absent. No glomerular, tubulointerstitial, or vascular lesions were present in renal parenchyma. In ZO rats moderate segmental increases in glomerular matrix, segmental collapse and obliteration of capillary lumina, and accumulation of hyaline were found. Such changes were frequently associated with fibrosis and synechial attachments to Bowman’s capsule. Tubulointerstitial injury was defined as inflammatory cell infiltrates, tubular dilation and/or atrophy, tubular casts or interstitial fibrosis, and tubular atrophy, tubular casts, and chronic inflammatory infiltrate were statistically significant increased in ZO rats (Table 2 and Figure 1).

**Table 2:** Histopathological results in male Zucker lean (ZL) and obese (ZO) rats at 8 months old.

Groups	ZL	ZO
Glomerular sclerosis	0 (0–0)	1 (1–1)^∗∗^
Glomerular hyperplasia	1 (1–1)	2 (2–2)^∗∗^
Mesangium increase	0.5 (0–1)	2 (2–2)^∗∗^
Capsular fibrosis	0 (0–0)	1 (1–1)^∗∗^
Glomerular cyst	0 (0–0)	1 (0–1)^∗^
Tubular vacuolization	1 (0–2)	2.5 (0–3)
Tubular atrophy	0 (0–0)	2 (2–2)^∗∗^
Tubular casts	0 (0–0)	2 (2–3)^∗∗^
Hyaline drops	1 (0–1)	2 (0–2)
Inflammation infiltrate	0 (0–0)	1 (1–1)^∗∗^

*Median and interquartilic range. ^∗^p < 0.05, ^∗∗^p < 0.001 vs. ZL (n = 10 in each group).*

### Plasma Variables and Creatinine Clearance at the End of the Study

Plasma urea and creatinine were decreased in ZO rats. Plasma glucose was not significantly different, probably because animals did not fast in order to obtain proper metabolic data. Creatinine clearance, expressed in absolute values or relative to g of kidney, was slightly increased in ZO rats, but did not reach statistical signification. These data are shown in Table 3.

**Table 3:** Plasma variables in male Zucker lean (ZL) and obese (ZO) rats at 8 months old.

Groups	ZL	ZO
Glucose (mg/dL)	249.7 ± 35.8	312.6 ± 35.0
Urea (mg/dL)	5.067 ± 0.065	3.833 ± 0.038^∗^
Creatinine (mg/dL)	0.577 ± 0.019	0.402 ± 0.075^∗^
CrCl (ml/min)	1.150 ± 0.061	2.033 ± 0.641
CrCl (ml/min/g kidney)	0.359 ± 0.025	0.485 ± 0.135

*Data are expressed as means ± SE. ^∗^P < 0.05 vs. the control group (n = 10 in each group). CrCl, creatinine clearance*.

### Time-Course of Urinary Biomarkers

**FIGURE 2:**
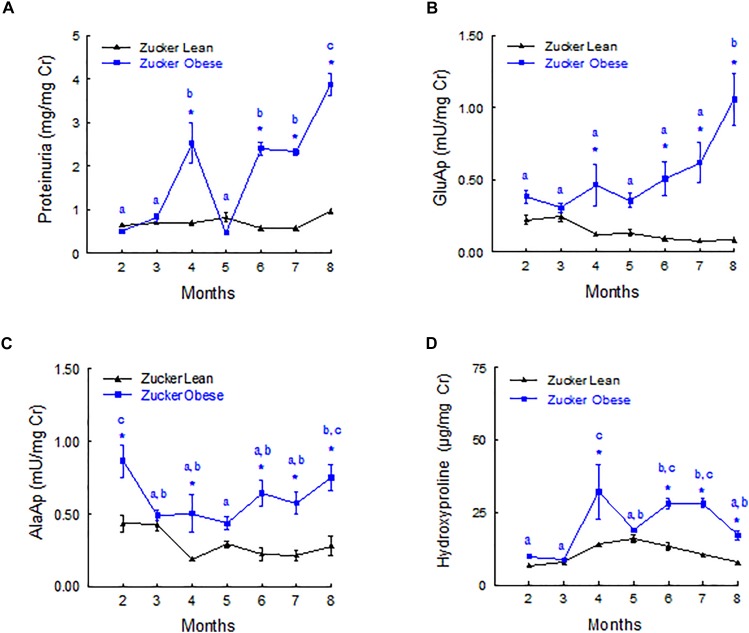
Time course of urinary biomarkers normalized by creatinine excretion. Proteinuria **(A)**, GluAP **(B)**, AlaAP **(C)**, and hydroxyproline **(D)** in male Zucker lean and obese rats. Data are means ± SEM. ^∗^*p* < 0.05 compared with Zucker lean rats (*n* = 10, each group). Means with the same letter (a, b, or c) indicates sets of means within there are no significant difference at *p* < 0.05.

Figure 2 shows the evolution of urinary biomarkers along the study. Proteinuria and GluAp were augmented in ZO rat at 4, 6, 7, and 8 months old, when expressed relative to creatinine excretion (mg/mg of Cr), showing a progressive time-related increase. AlaAp was increased in ZO rats at 2, 4, 6, 7, and 8 months old, showing a U-shaped pattern of evolution. Hydroxyproline was significantly increased in ZO rats at 2, 4, 6, 7, and 8 months old.

### Plasma and Renal Aminopeptidasic Activities

GluAp and AlaAp enzymatic activities remained unchanged in the kidney of ZO rats at the end of the experiment. In plasma, there was a slight increase of GluAp activity in ZO rats, whereas plasmatic AlaAp activity remained unchanged (Table 4). The ratio of augmentation of GluAp activity in urine from ZO vs. ZL rats at 8 months old was 11.8 whereas the plasmatic ratio of augmentation was 1.43. Urinary AlaAp activity was nearly three times increased, even though there was no variation in its plasmatic activity.

**Table 4:** Plasmatic, renal and urinary enzymatic activities of GluAp and AlaAp at 8 months old in male Zucker lean (ZL) and Zucker obese (ZO) rats.

Groups	ZL	ZO
**Plasma**		
GluAp (mU/ml)	3.03 ± 0.19	4.34 ± 0.32^∗^
AlaAp (mU/ml)	2.23 ± 0.04	2.45 ± 0.17
**Kidney**		
GluAp (mU/mg protein)	8.16 ± 0.85	8.78 ± 0.70
AlaAp (mU/mg protein)	11.1 ± 1.19	11.5 ± 0.97
**Urine**		
GluAp (mU/mg creatinine)	0.09 ± 0.02	1.06 ± 0.18^∗∗^
AlaAp (mU/mg creatinine)	0.28 ± 0.07	0.75 ± 0.09^∗∗^

*Data are expressed as means ± SE. ^∗^p < 0.01, ^∗∗^p < 0.001 vs. ZL group (n = 10 in each group)*.

### Plasma, Urinary and Renal Klotho

**FIGURE 3:**
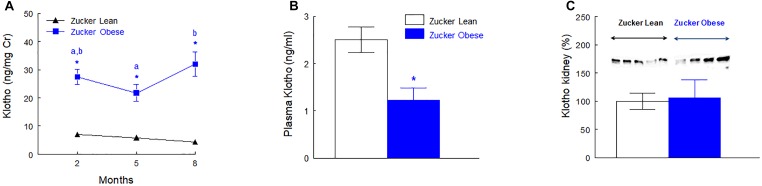
Urinary excretion of Klotho normalized by creatinine excretion **(A)**, plasma levels of Klotho **(B)** at the end of the experiment (8 months old) and renal protein abundance of Klotho **(C)** in male Zucker lean and obese rats. Data are means ± SEM. ^∗^*p* < 0.05 compared with Zucker lean rats (*n* = 10, each group). Means with the same letter (a or b) indicates sets of means within there are no significant difference at *p* < 0.05.

The urinary excretion of Klotho was higher in ZO rats at 2, 5, and 8 months old when expressed as total daily excretion (ng/rat/day), urinary concentration (ng/ml) or relative to creatinine excretion (ng/mg Cr). The first two ways of expression are not presented. Plasma Klotho concentration was reduced to the half (*p* < 0.01) in ZO rats when compared with ZL group. The protein abundance of Klotho in renal tissue was similar in ZO and ZL rats. These data are shown in Figure 3 and Supplementary Figure S1.

**FIGURE 4:**
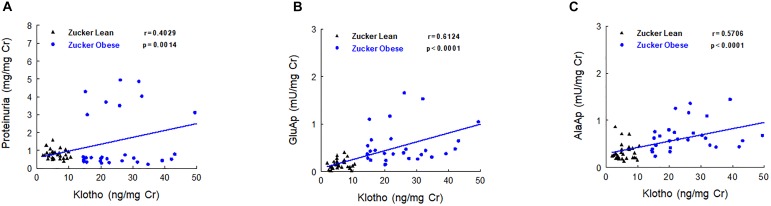
Correlations between urinary Klotho and proteinuria **(A)**, urinary GluAp **(B)** and urinary AlaAP **(C)** excreted at 2, 5, and 8 months old, when all animals (lean and obese Zucker rats) were pooled in a common regression line.

Urinary Klotho showed positive correlations with proteinuria, urinary GluAp and urinary AlaAP excreted at 2, 5, and 8 months old, when all animals (ZL and ZO rats) were pooled in a common regression line, reaching the strongest correlation with urinary GluAp (Figure 4).

### Fibrosis Related Variables

**FIGURE 5:**
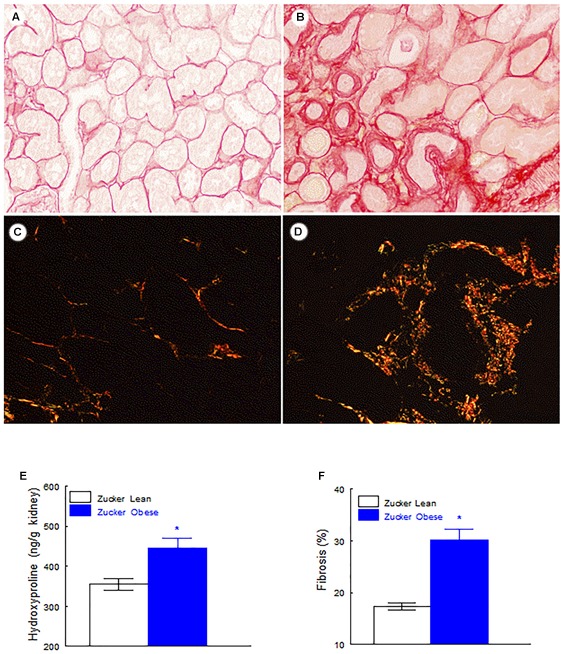
Fibrosis related variables. Renal level of fibrosis in Zucker lean rats **(A,C)** and Zucker obese rats **(B,D)**, Note the significant increase of tubulointerstitial fibrosis in obese rats in comparison with lean rats (**A,B** Sirius red stain with light microscope, **C,D** Sirius red stain with polarized light, original magnification 20×). Renal hydroxyproline levels **(E)** and percentage of renal fibrosis **(F)**. Data are means ± SEM. ^∗^*p* < 0.01 compared with Zucker lean rats (*n* = 10, each group).

Renal hydroxyproline content was increased in renal tissue of obese rats and morphometrical quantification with Sirius red stain and polarized light of renal cortex showed a significant increase of tubulointerstitial fibrosis in Zucker obese rats (*p* < 0.001). These data are displayed in Figure 5.

### Correlation Studies

Table 5 shows a multiple regression analysis that included GluAp, AlaAp, proteinuria, Klotho, and hydroxyproline excretion normalized per mg of creatinine in urine collected at 2 months old, GluAp, AlaAp, and Klotho or their combination were the markers that better predicted all renal lesions quantified at the end of the experiment. Results of multiple regression analysis at 5 months old were very similar to that of 2 months. GluAp, AlaAp, and Klotho were the best predictors of renal lesions (Table 6).

**Table 5:** Equation of the fitted model, correlation coefficient (*r*) and *p*-value of the multiple linear regression between urinary markers measured at 2 months and renal lesions at the end of the experiment (8 months old) in Zucker lean and Zucker obese rats (*n* = 20).

2 months	Equation of the fitted model	*r*	*p*
Glomerular esclerosis	*Y* = -0.278 + 0.484AlaAp + 0.024Klotho	0.7993	0.0002
Glomerular hyperplasia	*Y* = 0.866 + 0.037Klotho	0.8660	<0.0001
Increased mesangium	*Y* = 0.265 + 0.054Klotho	0.7869	<0.0001
Capsular fibrosis	*Y* = -0.543 + 1.049AlaAp + 0.018Klotho	0.8607	<0.0001
Glomerular cysts	*Y* = 0.0342 + 0.0241Klotho	0.5712	0.0085
Tubular vacuolization	*Y* = -0.0155 + 2.18AlaAp	0.6204	0.0035
Tubular atrophy	*Y* = -0.444 + 1.684GluAp + 0.0586Klotho	0.8774	<0.0001
Casts	*Y* = -0.604 + 3.18GluAp + 0.0517Klotho	0.7858	0.0003
Hyaline drops	*Y* = 0.019 + 1.82AlaAp	0.6031	0.0049
Inflammation	*Y* = -0.147 + 0.0404Klotho	0.7117	0.0004
Interstitial fibrosis (SR)	*Y* = 0.414 + 2.53GluAp + 0.0931Klotho	0.7418	0.0011
Interstitial fibrosis (%)	*Y* = 9.501 + 29.6GluAp + 0.3055Klotho	0.8507	<0.0001
Renal Hyp	*Y* = 313 + 134.5AlaAp	0.6180	0.0037

*GluAp, glutamyl aminopeptidase; AlaAp, alanyl aminopeptidase; Hyp, hydroxyproline; SR, Sirius red*.

**Table 6:** Equation of the fitted model, correlation coefficient (*r*) and *p*-value of the multiple linear regression between urinary markers measured at 5 months and renal lesions at the end of the experiment (8 months old) in Zucker lean and Zucker obese rats (*n* = 20).

5 months	Equation of the fitted model	*r*	*p*
Glomerular esclerosis	*Y* = -0.162 + 1.16GluAp + 0.0238Klotho	0.7981	0.0002
Glomerular hyperplasia	*Y* = 0.887 + 1.07GluAp + 0.0256Klotho	0.8027	0.0002
Increased mesangium	*Y* = 0.520 + 0.0494Klotho	0.6327	0.0028
Capsular fibrosis	*Y* = -0.178 + 0.0456Klotho	0.8048	<0.0001
Glomerular cysts	*Y* = 0.117 + 0.0242Klotho	0.5059	0.0229
Tubular vacuolization	*Y* = 0.501 + 0.0652Klotho	0.5660	0.0093
Tubular atrophy	*Y* = -0.070 + 2.26GluAp + 0.0431Klotho	0.7777	0.0004
Casts	*Y* = -0.182 + 2.95GluAp + 0.0516Klotho	0.7831	0.0003
Hyaline drops	*Y* = 0.607 + 0.0430Klotho	0.4354	0.0550
Inflammation	*Y* = -0.142 + 0.0502Klotho	0.7815	<0.0001
Interstitial fibrosis (SR)	*Y* = 1.45 + 0.0363Klotho	0.5113	0.0212
Interstitial fibrosis (%)	*Y* = 7.56 + 44.66AlaAp	0.7023	0.0006
Renal Hyp	*Y* = 326 + 5.38Klotho	0.7525	0.0001

*GluAp, glutamyl aminopeptidase; AlaAp, alanyl aminopeptidase; Hyp, hydroxyproline; SR, Sirius red*.

At 8 months old, proteinuria, hydroxyproline, AlaAp, and Klotho were the markers with the best correlations with renal lesions (Table 7). Moreover, we also found correlations with renal lesions at 2, 5, and 8 months old when urinary markers were normalized by total daily excretion, and even when they were expressed in urine concentration.

**Table 7:** Equation of the fitted model, correlation coefficient (*r*) and *p*-value of the multiple linear regression between urinary markers measured at 8 months and renal lesions at the end of the experiment (8 months old) in Zucker lean and Zucker obese rats (*n* = 20).

8 months	Equation of the fitted model	*r*	*p*
Glomerular esclerosis	*Y* = -0.356 + 0.0126Klotho + 0.0465Hyp	0.8636	<0.0001
Glomerular hyperplasia	*Y* = 0.787 + 0.2265Prot + 0.0090Klotho	0.9541	<0.0001
Increased mesangium	*Y* = 0.2661 + 0.2409Prot + 0.0193Klotho	0.8032	0.0001
Capsular fibrosis	*Y* = -0.118 + 0.0313Klotho	0.8871	<0.0001
Glomerular cysts	*Y* = -0.158 + 0.0491Hyp	0.5727	0.0083
Tubular vacuolization	*Y* = 0.327 + 2.08AlaAp	0.5799	0.0074
Tubular atrophy	*Y* = -0.238 + 0.4153Prot + 0.0169Klotho	0.9230	<0.0001
Casts	*Y* = -0.4564 + 0.7042Prot	0.9346	<0.0001
Hyaline drops	*Y* = 0.2728 + 1.8015AlaAp	0.5842	0.0068
Inflammation	*Y* = -0.602 + 0.0181Klotho + 0.0664Hyp	0.9181	<0.0001
Interstitial fibrosis (SR)	*Y* = 1.17 + 0.322Prot	0.6805	0.0010
Interstitial fibrosis (%)	*Y* = 12.9 + 4.46Prot	0.8691	<0.0001
Renal Hyp	*Y* = 310 + 112AlaAp + 1.7623Klotho	0.8154	0.0001

*AlaAp, alanyl aminopeptidase; Prot, proteinuria; Hyp, hydroxyproline; Hyp, hydroxyproline; SR, Sirius red*.

**FIGURE 6:**
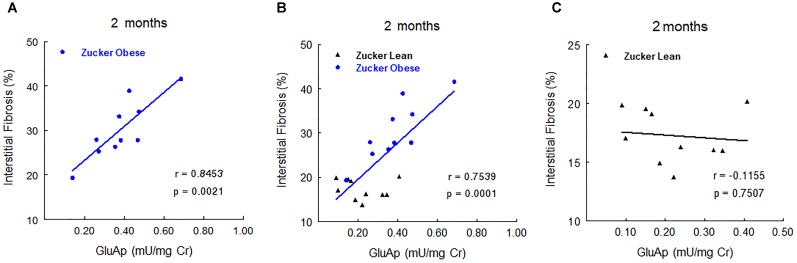
Predictive correlations of urinary GluAp activity at 2 months old with interstitial fibrosis at the end of the experiment in ZO rats (**A**, *n* = 10), in ZL and ZO rats (**B**, *n* = 20) and in ZL rats (**C**, *n* = 10).

In order to study the prognostic value of urinary markers in ZO group, we carried out a multiple regression analysis between urinary markers at 2, 5, and 8 months old and the two continuous variables at the end of the experiment: renal fibrosis and renal hydroxyproline. At 2 months old, GluAp urinary activity was the marker that showed the highest predictive correlation with renal fibrosis in ZO group (Figure 6), indicating that this marker can have an added value as an early prognostic marker of the extent of fibrosis. Inclusion of ZL rats in the analysis made the correlation to be weaker, and no correlation was found if analysis was carried out using only ZL rats (Figure 6). These results can explain that urinary activity of GluAp at 2 months old was not found to be significantly increased in ZO rats, because some ZO rats have a low urinary activity, but they are the ones that exhibit lower levels of fibrosis at the end of the experiment. We did not find significant correlations with renal fibrosis for any other marker or their combinations at 5 or 8 months old when only ZO rats were used.

**FIGURE 7:**
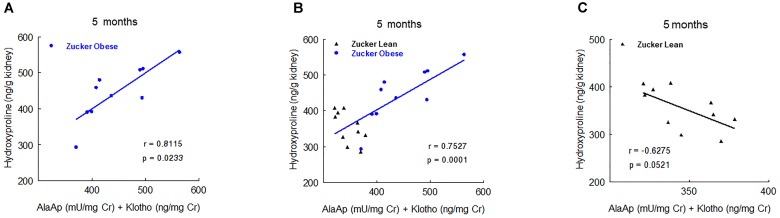
Predictive correlations of the combination of AlaAp and Klotho at 5 months old with renal hydroxyproline content at the end of the experiment in ZO rats (**A**, *n* = 10), in ZL and ZO rats (**B**, *n* = 20) and in ZL rats (**C**, *n* = 10).

**FIGURE 8:**
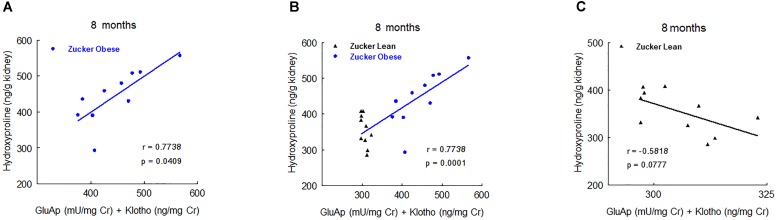
Predictive correlations of the combination of GluAp and Klotho at 8 months old with renal hydroxyproline content at the end of the experiment in ZO rats (**A**, *n* = 10), in ZL and ZO rats (**B**, *n* = 20) and in ZL rats (**C**, *n* = 10).

Nevertheless, at 5 and 8 months old we found significant correlations with renal hydroxyproline content. At 5 months old, the combination of urinary AlaAp activity and Klotho showed the maximal prognostic correlation (Figure 7), while the combination of GluAp and Klotho was the marker that best fitted with renal hydroxyproline content at 8 months old (Figure 8).

Altogether, these results demonstrate that GluAp, AlaAp, and Klotho are related with the severity of renal lesions even when animals are 2 months old, and they can serve not only to differentiate these lesions between ZO and ZL rats but also to establish a prognostic of these lesions inside ZO group.

## Discussion

This study clearly shows for first time that obesity of Zucker rats is associated to increased urinary glutamyl and alanyl aminopeptidasic activities and to increased urinary excretion of Klotho, changes that were detected as early as 2 months old. This study also shows that urinary aminopeptidases and urinary Klotho correlates with the morphological changes observed in the renal injury of the ZO rats analyzed at the end of the experimental period. Moreover, renal hydroxyproline content and tubulointerstitial fibrosis were increased in the renal tissue of obese rats.

The present paper shows that kidneys from ZO rats at 8 months old developed mild focal and segmental glomerulosclerosis as well as moderate tubulointerstitial injury, data that are in consonance with those reported by other authors in this type of experimental obesity (Kasiske et al., 1985, 1992; Magil, 1995; Coimbra et al., 2000; Gassler et al., 2001; D’Agati et al., 2016). It is interesting to note that despite to these histopathological findings of renal injury in ZO rats, the classic biomarkers of renal disease –plasma urea and creatinine and creatinine clearance are normal or even better than normal in our study, as well as in the literature (Lavaud et al., 1996; Coimbra et al., 2000; Gassler et al., 2001), indicating that structural renal changes might be present before the manifestation of renal disease and that the classic biomarkers of renal disease are not useful tools for the evaluation of obesity induced renal damage. In fact, Coimbra et al. (2000) reported that plasma creatinine and urea remained normal during the observation period until week 40, and they started to increase at a later stage of renal injury, when Zucker obese rats were 60 weeks old.

The data concerning urinary aminopeptidasic activities are in consonance with previous papers, where we showed that urinary activity of these enzymes were associated to the development of renal damage in hypertensive hyperthyroid rats under high salt intake, showing that urinary aminopeptidasic activity correlates with the degree of renal injury but not with plasma renin activity or angiotensin II plasma levels (Pérez-Abud et al., 2011) and in cisplatin-treated rats (Quesada et al., 2012). In these studies, AlaAp and GluAp activities also were manifested as early and predictive biomarkers of the presence and severity of renal injury (Quesada et al., 2012; Montoro-Molina et al., 2015). These enzymes reach urine from damaged tubular cells, and they can detect proximal tubular injury regardless of the glomerular filtration status (Quesada et al., 2017).

Data obtained from the Prevention of Renal and Vascular End Stage Disease (PREVEND) study demonstrate that albuminuria augmented during the development of diabetic kidney disease and that, in addition, is a marker of the progression of diabetes (Brantsma et al., 2005). In our study, ZO rats also shows increased proteinuria that started at 4 months old, at 2 months of the observational period. However, other authors that also studied the time course of proteinuria in ZO rats reported the appearance of proteinuria in later stages of this type of obesity, e.g., 10 months of age (Michel et al., 1997; Coimbra et al., 2000; Gassler et al., 2001). Very recently, Mima et al. (2018) have observed that the appearance of albuminuria can be accelerated when ZO rats are fed with high-fat chow.

Proteinuria from the classic point of view is secondary to alterations of the glomerular barrier membrane, in fact podocyte injury underlies the progression of focal segmental glomerulosclerosis in the fa/fa Zucker rat (Gassler et al., 2001), but now it is also well known that, under physiological conditions, 7–9 g/day of proteins can cross the glomerular barrier membrane and appeared in primary urine in humans (Haraldsson and Sörensson, 2004). These proteins are uptaked by tubular cells (Haraldsson et al., 2008) by the megalin-cubulin complex (Christensen and Birn, 2001) and degraded and fragmented in the tubule (Eppel et al., 2001). Thus, lysine has been administered to produce proteinuria by inhibiting tubular cells (Tencer et al., 1998). Hence, it is possible that, at least, a part of the proteinuria observed in ZO rats may arise from a tubular injury, as indicated the increased urinary levels of aminopeptidases -localized in the brush border of tubular cells- that precede the appearance of proteinuria. Therefore, an early tubular injury could determine a defective tubular uptake of proteins. In consonance with this, Osicka et al. (2000) reported that the proteinuria that occurs with diabetic nephropathy reflects defects in the tubular system.

This study shows increased urinary levels of Klotho, a reduction in plasma and normal values of protein abundance in the renal tissue. These last data agree with the results of Lorenzi et al. (2010) that found no differences in klotho mRNA levels between obese Zucker and control rats, neither in kidneys nor in other organs; and that Klotho protein levels tested by Western blot were similar in kidneys from ZO and control rats. However, the present results contrast with the decreased renal gene expression of Klotho observed in various animal models of vascular and metabolic diseases (Nagai et al., 2000). Moreover, our results also contrast with previous reports in other renal diseases, where plasma, renal and urinary Klotho go in the same direction, considering that serum and urinary Klotho can be surrogate markers for renal Klotho production (Hu et al., 2011).

The possible explanation for the absence of uniformity of our Klotho data is that tubular injury determines an increased urinary excretion of Klotho as happens with aminopeptidases that are released to urine. This wasting of Klotho through the urine, might contributes to the reduced plasma level, since urinary αKlotho, at least in part, may arise from the plasma. Thus, exogenously injected labeled αKlotho was detected in the urine of rats (Hu et al., 2016). In support of our hypothesis is the positive correlation between urinary aminopeptidases and urinary Klotho, indicating that Klotho and aminopeptidases probably are augmented in urine due to the presence of damaged tubular cells.

As reported in the Introduction section, plasma soluble Klotho may operate as an endocrine agent on distant organs (Hu et al., 2013, 2015), with many cardiovascular and protective effects (Hu et al., 2013, 2015; Lu and Hu, 2017). Thus, the reduced plasma levels of Klotho might contribute to the cardiovascular and renal abnormalities observed in ZO rats, since reduced circulating Klotho also have been reported in other renal (Hu et al., 2010b, 2011, 2012; Akimoto et al., 2012; Scholze et al., 2014) or CVDs (Wang and Sun, 2009; Yu et al., 2010), including diabetes in humans and mice (Zhao et al., 2011; Asai et al., 2012).

Urinary hydroxyproline excretion has been related in previous works with the development of different renal lesions including fibrosis in a murine model of nephrotoxicity evoked with melamine and cyanuric acid (Schnackenberg et al., 2012). In our study, urinary hydroxiproline was increased in ZO rats from two 2 months old, although our data demonstrate that the excretion of Klotho, GluAp, and AlaAp activities at 2 and 5 months old correlate better than hydroxyproline even with renal fibrosis or renal hydroxyproline content. At 8 months old, urinary hydroxyproline excretion takes relevance as a diagnostic marker of glomerular sclerosis, glomerular cysts and inflammation, but not for renal fibrosis or renal hydroxyproline content. Furthermore, in our study, urinary hydroxyproline was not a prognostic marker, because it did not show any correlation with these variables in ZO rats. This lack of correlation might be due to the fact that urinary hydroxyproline mainly reflects plasmatic levels of this amino acid, because it is freely filtered in glomerulus, although hydroxyproline derived from collagen of renal tissue can also contribute to increase its content in urine, explaining the higher excretion of this marker in ZO rats when compared with ZL rats.

The correlation studies shown in Tables 5, 6 demonstrate that GluAp, AlaAp, and Klotho are early diagnostic markers of renal lesions in ZO rats. Proteinuria and hydroxyproline can be considered delayed diagnostic markers because their contribution to diagnosis starts later, probably when glomerular and fibrotic lesions are more extended. It is interesting to note that all urinary markers were normalized per creatinine excretion because it allows analyzing spot samples in clinical practice without the needing of collecting 24-h urine and it is the most used way to quantify urinary markers, but we have obtained similar correlations when markers were expressed in total daily excretion or even in urinary concentration.

Another relevant result is that GluAp, AlaAp and Klotho are related not only with diagnosis but also with prognosis of renal lesions in Zucker obese rats. Results reported in Figures 6–8 show strong predictive correlations with percentage of renal fibrosis or with renal hydroxyproline content at the end of the experiment, indicating that an early increased excretion of these markers is related with a higher later extent of fibrosis in ZO rats. Finally, we also want to pointed out that the correlations observed between tubular urinary biomarkers and the morphological signs of renal injury, do not establish any causal relationship among them.

## Conclusion

Urinary aminopeptidases and Klotho are early diagnostic biomarkers of renal injury, and urinary levels of these biomarkers are also related with the extent of renal fibrosis in Zucker obese rats. Measurements of GluAp, AlaAp, and Klotho may represent a novel, specific and non-invasive diagnostic approach to assess kidney fibrosis and may have a potential use not only in diagnosis but also in prognosis of obesity-associated renal lesions.

## Author Contributions

AO, FV, and RW: conception and design of the experiments. AL-C, AQ, FO’V, NM-M, SM-M, and RW: collection, analysis and interpretation of the data. FO’V, AO, FV, and RW: drafting the article or revising it critically for important intellectual content. All authors reviewed and approved the final version of the manuscript.

## Conflict of Interest Statement

RW, FV, and AO are co-authors of the patent “Glutamyl aminopeptidase as a marker of renal damage” with publication number 2382960. There is not any other relevant declaration related to employment, consultancy, patents, products in development or modified products. The remaining authors declare that the research was conducted in the absence of any commercial or financial relationships that could be construed as a potential conflict of interest.
