# Supplementary figures and images for: Klotho and Aminopeptidases as Early Biomarkers of Renal Injury in Zucker Obese Rats
Source: Front Physiol. 2018 Nov 13;9:1599. doi: 10.3389/fphys.2018.01599 (PMC6243116; doi:10.3389/fphys.2018.01599)

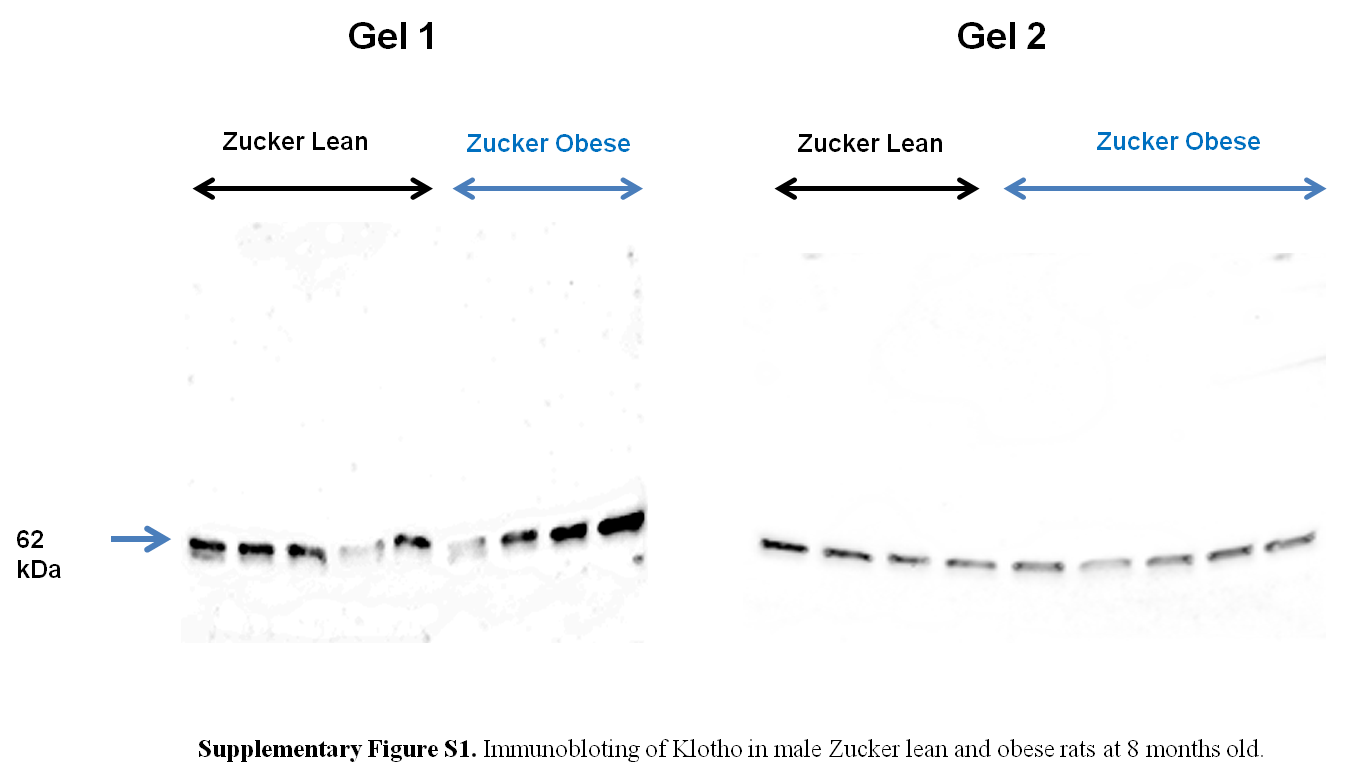

Supplement: Supplementary file 1 [file Image_1.tif]
